# Trp–His covalent adduct in bilirubin oxidase is crucial for effective bilirubin binding but has a minor role in electron transfer

**DOI:** 10.1038/s41598-019-50105-3

**Published:** 2019-09-23

**Authors:** Tomáš Kovaľ, Leona Švecová, Lars H. Østergaard, Tereza Skalova, Jarmila Dušková, Jindřich Hašek, Petr Kolenko, Karla Fejfarová, Jan Stránský, Mária Trundová, Jan Dohnálek

**Affiliations:** 1 Institute of Biotechnology of the Czech Academy of Sciences, v.v.i., Průmyslová 595, 252 50 Vestec, Czech Republic; 2 Faculty of Nuclear Sciences and Physical Engineering, Czech Technical University in Prague, Břehová 7, 115 19 Praha 1, Czech Republic; 3 Novozymes A/S, Krogshoejvej 36, 2880 Bagsvaerd, Denmark

**Keywords:** Enzyme mechanisms, X-ray crystallography

## Abstract

Unlike any protein studied so far, the active site of bilirubin oxidase from *Myrothecium verrucaria* contains a unique type of covalent link between tryptophan and histidine side chains. The role of this post-translational modification in substrate binding and oxidation is not sufficiently understood. Our structural and mutational studies provide evidence that this Trp396–His398 adduct modifies T1 copper coordination and is an important part of the substrate binding and oxidation site. The presence of the adduct is crucial for oxidation of substituted phenols and it substantially influences the rate of oxidation of bilirubin. Additionally, we bring the first structure of bilirubin oxidase in complex with one of its products, ferricyanide ion, interacting with the modified tryptophan side chain, Arg356 and the active site-forming loop 393-398. The results imply that structurally and chemically distinct types of substrates, including bilirubin, utilize the Trp–His adduct mainly for binding and to a smaller extent for electron transfer.

## Introduction

Bilirubin oxidase (*Mv*BOx; EC 1.3.3.5) from the ascomycete plant pathogen *Myrothecium verrucaria* (*Albifimbria verrucaria*) is a member of the blue multicopper oxidase family (MCO). MCOs are capable of oxidizing various organic and/or inorganic substrates and reducing oxygen to water without release of reactive oxygen species^1–6^. *Mv*BOx, composed of 534 amino acid residues, consists of three cupredoxin-like domains with four copper ions forming two active sites^7,8^. These copper ions can be divided into three classes according to their spectroscopic properties^9,10^. One copper ion is of type I (T1Cu) and is present at the so called T1Cu site near the protein surface. Coordination of this copper ion is responsible for the distinctive blue color of *Mv*BOx and all MCOs (absorption at 600 nm) and for oxidation of substrates with the Cu^2+^ ion being an electron acceptor^9–11^. T1Cu is connected with the trinuclear cluster (TNC), composed of one type II (T2Cu) and two type III (T3Cu) copper ions, via a conserved His–Cys–His motif (serving as electron transfer path). The binuclear T3Cu site with a bridging hydroxyl or dioxygen is responsible for a characteristic shoulder at approximately 330 nm in UV–VIS absorption spectrum. At the TNC one molecule of O_2_ is reduced to two molecules of water using four electrons supplied by the T1Cu site^9–18^.

*Mv*BOx can oxidize a variety of substrates (Fig. S1) including bilirubin, 2,2′-azino-bis(3-ethylbenzothiazoline-6-sulfonic acid) (ABTS), substituted phenols, or ferrocyanide ([Fe(CN)_6_]^4−^), with different pH optima for different classes of substrates^6,19^. *Mv*BOx can be utilized for a range of purposes. In medicine, it is used for diagnostics of bilirubin in serum^19–21^, in biotechnology for decolorization of synthetic dyes or detoxification of the environment^19,22,23^. It also shows a potential for use in biosensors and biofuel technology^24–33^.

The mechanism of dioxygen reduction inside the TNC as well as the electron transfer path between the T1Cu site and the TNC are very similar within the MCO family and are well understood. They were intensively studied using biochemical, structural, and computational methods^12,16,34–36^. However, the mechanism of substrate binding and oxidation at the T1Cu site varies among MCOs and is known only for several representatives (e.g. laccases^37–39^). In most laccases and many other MCOs the T1Cu site can be directly accessed by substrate, usually with a direct contact (or with a very short distance) of the oxidized moiety to one of the T1Cu-coordinating histidine side chains. In *Mv*BOx the T1Cu site is separated from bulk solvent by additional amino acid residues, including Trp396. This raises questions regarding the separation of the substrate binding site and the T1Cu site in relation to the enzyme function.

Five structures of *Mv*BOx have been published so far^7,8,40^, however, they do not show binding of any ligands besides water molecules in the proximity of the T1Cu site. Here we present the first complex of *Mv*BOx with a product, ferricyanide ion ([Fe(CN)_6_]^3−^), binding close to the T1Cu site, together with the structure-function analysis of the protein layer between the T1Cu site and the identified substrate binding site containing a natural post-transitional modification, the covalent crosslink between T1Cu-bound His398 and Trp396^40^. We examined its role in the reaction mechanism by mutagenesis connected with structure-function analysis.

## Results

### Structure of MvBOx wild type from acidic pH

**Figure 1:**
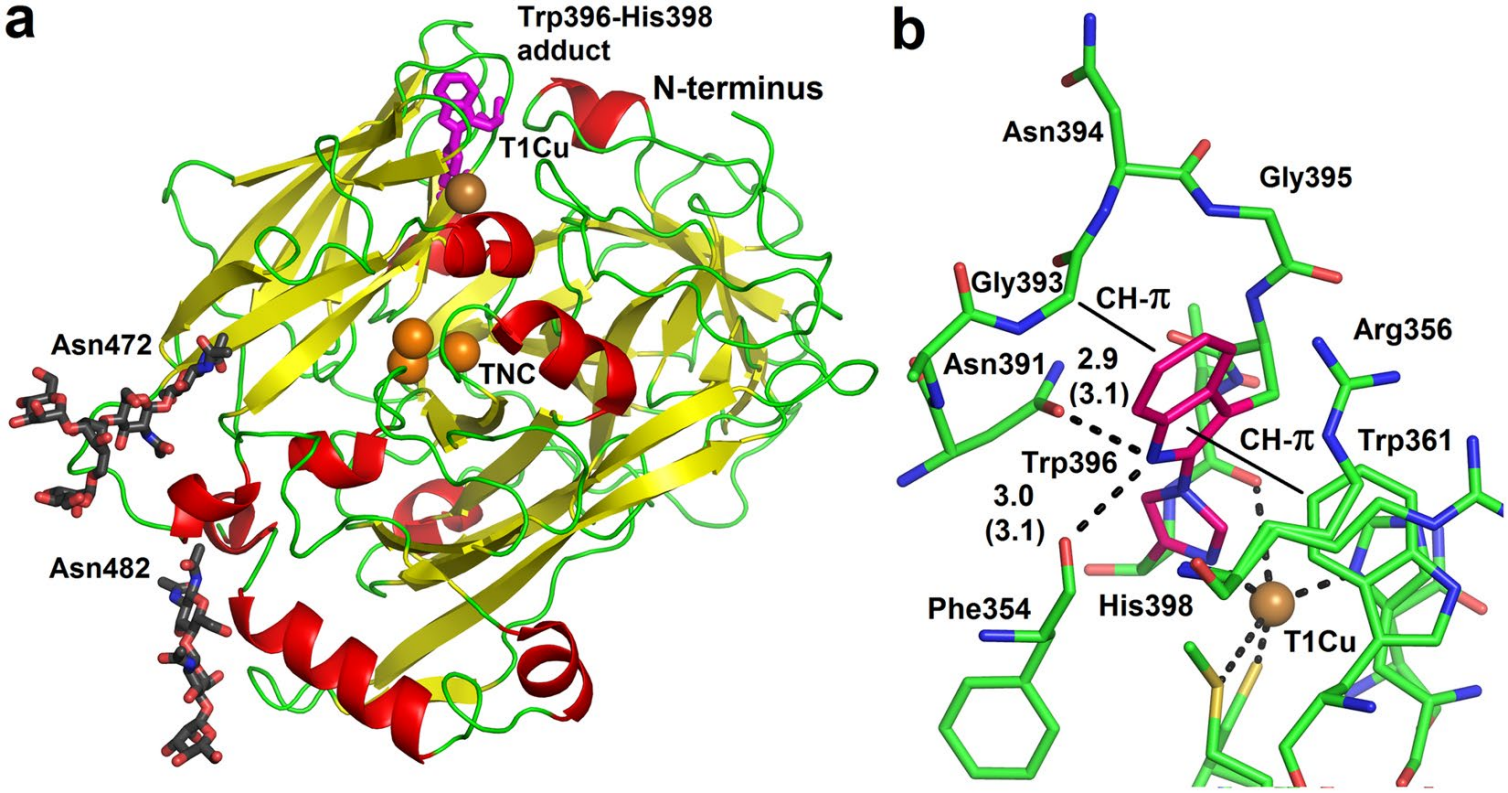
(**a**) Structure of MvBOxWT from strongly acidic condition. The structure (6I3J) is shown in secondary structure representation (helices are colored red, β–strands yellow, loops green). Copper ions are shown as orange spheres. The Trp396–His398 adduct (shown as magenta sticks), oligosaccharides modifying asparagine side chains (black sticks), the N-terminus, T1 copper and the trinuclear copper cluster are labeled. (**b**) Chemical environment of the Trp–His adduct in *Mv*BOx. Hydrogen bond distances are given in Ångströms. Values in parentheses are for chain B of the structure 6I3J. The CH – π interactions of Trp396 are marked. The indole–imidazole moiety of the adduct is shown with carbon colored magenta. T1Cu is shown as orange sphere. Molecular graphics were created using PyMOL (Schrödinger, LLC).

The crystal structure of *Mv*BOx wild type (*Mv*BOxWT) in complex with ferricyanide (PDB ID 6I3J; WT:FECN) was obtained from a strongly acidic crystallization condition (pH 3.1). *Mv*BOx crystallized in the space group *F*222 with two monomers in the asymmetric unit (ASU). Nevertheless, the protein fold is very similar to the previously reported structures of *Mv*BOx (Fig. 1a), one from a basic condition with a positional r.m.s.d. of 533 C^α^ atoms of 0.30 Å (PDB ID 2XLL, crystallization at pH 8.7, space group *P*1, four monomers in ASU^8^), and the second one from an acidic condition (PDB ID 6IQZ, pH 5, space group *C*2, one monomer in ASU^40^) with an r.m.s.d. of 0.25 Å. The structures were superimposed using the Secondary structure matching algorithm (SSM) in Coot^41^. Glycosylation of *Mv*BOx in the strongly acidic condition (at Asn472 and Asn482) is preserved and was modelled (Fig. 1a). Conformations of all residues (including the side chains) around the T1Cu site are basically the same in all three structures (Fig. S2).

### Trp396–His398 crosslink

The covalent crosslink between the Trp396 and His398 side chains was initially identified due to the observed close contact between Trp396-C^δ1^ and His398-N^ε2^ supported by the electron density (Fig. 1). The existence of the Trp396–His398 crosslink was confirmed afterwards by mass spectrometry observing an ion at m/z 649.8327 (4+) corresponding to the modified WELINAGNGWTHPIHIHLVDFK peptide (Fig. S3). The correct assignment of the peptide ion was confirmed by collision induced dissociation.

Refinement of this type of covalent link required a proper definition of its geometry. There were several examples of X-ray structures of small molecules in the Cambridge Structural Database (CSD, see Materials and Methods for details) containing this type of bond and suitable for extraction of geometrical restraints. The initial refinement of the structure imposing only a covalent link restraint with the target value of the distance between Trp396-C^δ1^ and His398-N^ε2^ of 1.41 Å led to small distortions of the planar side chains of both residues and a slight deviation of the new bond from the planes of both side chains (with opposite signs in chains A and B). Therefore, co-planarity of the bond with each side chain (independently) was further restrained, with target values of bond distances and angles in the nearest vicinity derived from the CSD entries. In this way the distance was refined to 1.42 Å and 1.40 Å (in chains A and B, respectively), following closely the set target values for the bond distance (1.41 Å) and the nearby environment. This approach led to a successful refinement of this moiety and to a good agreement with electron density without any difference peaks at this site.

The Trp396 side chain of the Trp–His adduct in *Mv*BOx is involved in several interactions (Fig. 1b). Trp396-N^ε1^ forms hydrogen bonds to the main chain oxygen of Phe354 and the side chain oxygen of Asn391. It is also involved in CH–π interactions with Gly393-C^α^ and the Trp361 side chain. The closest surroundings of Trp–His in *Mv*BOx is further formed by the side chain of Arg356 and the main chain atoms of the 393–395 loop (Gly393, Asn394, Gly395).

### Functional mutants of *Mv*BOx Trp396

To analyze the role of the Trp396–His398 adduct, three functional variants of *Mv*BOx were designed and prepared. Trp396 was mutated to alanine (*Mv*BOxW396A) in order to enable direct solvent access to His398, to phenylalanine (*Mv*BOxW396F) in order to introduce an aromatic residue not capable of crosslink formation, and to aspartic acid (*Mv*BOxW396D) in order to disrupt this site by introducing negative charge. All three *Mv*BOx variants have the same composition of the secondary structure elements (overall structure) as the wild type (See Fig. S4 for CD spectrometry results) and all have the T1Cu site preserved, which is clear from the blue color of their solutions (See Fig. S5 for UV-VIS spectra).

### Structures of *Mv*BOx mutants Trp396Ala and Trp396Phe from acidic pH

Similarly to the structure of WT:FECN, also the crystal structure of *Mv*BOxW396A in complex with ferricyanide (PDB ID 6I3K; W396A:FECN) and the structure of *Mv*BOxW396F without any ligand near the T1Cu site (PDB ID 6I3L; W396F) were obtained from the same acidic crystallization condition. Both proteins crystallized in the space group *F*222. In neither mutant the protein fold was affected by the mutation. The positional r.m.s.d. of 533 C^α^ atoms between the structures WT:FECN (PDB ID 6I3J, chain A) and W396A:FECN (PDB ID 6I3K, chain A) or W396F (PDB ID 6I3L, chain A) was 0.23 Å and 0.21 Å, respectively. The surroundings of the T1Cu site were also unaffected (Figs S6 and S7).

### Coordination of T1Cu

**Figure 2:**
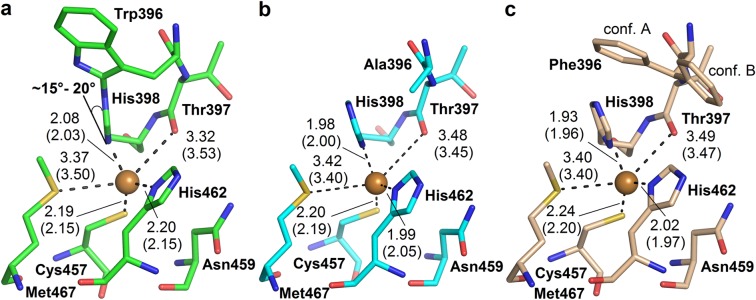
Coordination of T1Cu: (**a)** in the structure of WT:FECN (PDB ID 6I3J, carbon green), **(b)** in W396A:FECN (PDB ID 6I3K, carbon light blue), and **(c)** in W396F (PDB ID 6I3L, carbon pale orange). Distances are given in Ångströms with 0.01 Å precision to support the discussion of the T1Cu environment changes. Values in parentheses are for chain B of the corresponding structure. T1 copper is shown as orange sphere. The Phe396 side chain in the structure W396F adopts two conformations marked conf. A and conf. B. Molecular graphics were created using PyMOL (Schrödinger, LLC).

The coordination of T1Cu in WT:FECN differs in two features when compared to those of MCOs without the Trp–His adduct (e.g. CotA^42^). The first difference lies in the fact that T1Cu is no longer in the imidazole plane of coordinating His398, but the histidine side chain is tilted by about 15–20°. The second difference is a longer coordination distance (~2.2 Å) between T1Cu and His462-N^δ1^ (Fig. 2). These features are present only in the structure WT:FECN (PDB ID 6I3J). In the structures of both mutants (PDB ID 6I3K and 6I3L) T1Cu is coordinated in the plane of the His398 side chain and the distance between T1Cu and His462-N^δ1^ is shorter (~2.0 Å) and similar with that in CotA or other MCOs.

### Binding of ferricyanide in the active site of *Mv*BOxWT and *Mv*BOxW396A

**Figure 3:**
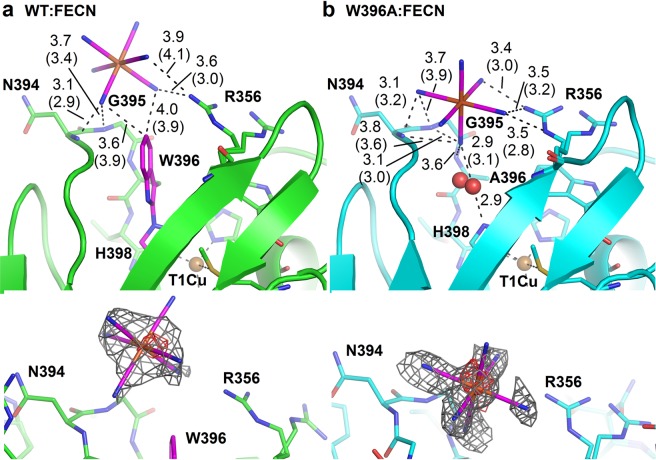
Binding of ferricyanide in the active site of *Mv*BOx wild type and its W396A mutant. (**a**) Binding of ferricyanide in OS1 of the WT:FECN structure (6I3J, carbon green). The ferricyanide ion and Trp–His adduct are shown with carbon colored magenta. (**b**) Binding of ferricyanide (magenta) in OS1 of the W396A:FECN structure (6I3K, carbon light blue). One water molecule (shown as red sphere, in two alternative positions) connects ferricyanide and His398. Interacting residues are marked. Distances are given in Ångströms. If values differ in chain A and B, they are given in parentheses for chain B of the corresponding structure. T1Cu is shown as orange sphere. The composite omit electron density map (2m*F*o*-*D*F*c) is shown as grey mesh and contoured at 1.0 σ level around the ferricyanide ion at the bottom of each panel. The map was calculated using *Phenix*^67^. Anomalous difference Fourier is shown as red mesh and contoured at 2.5 σ level around iron. Molecular graphics were created using PyMOL (Schrödinger, LLC).

A ferricyanide ion was identified in the active site of both WT:FECN and W396A:FECN (PDB ID 6I3J and 6I3K, respectively) using difference electron density (m*F*_o_-D*F*_c_) after the phase problem solution. Its presence was confirmed by a peak in anomalous difference Fourier at the iron atom and composite omit map (Fig. 3). Complexes were prepared by soaking crystals in the solution containing ferrocyanide (substrate, see Materials and Methods section). During this process crystals of both *Mv*BOxWT and *Mv*BOxW396A gradually changed their blue color to transparent which proved the reduction of the T1Cu site in parallel with ongoing oxidation of ferrocyanide in the crystals (Supplementary video sequences 1 and 2).

In WT:FECN, the ferricyanide ion was bound in close proximity of the Trp396–His398 adduct, interacting with the main chain nitrogen atoms of Asn394 and Gly395 through one of its cyanide moieties and with the Arg356 side chain through two adjacent cyanide moieties (Fig. 3a). The shortest distance observed between ferricyanide and T1Cu is 11.4 Å (Fig. S8a). This is well within the 14 Å limit identified as the boundary for efficient electron transfer in proteins^43^. Moreover, ferricyanide in this position is not involved in any symmetry (crystal-induced) contacts, which supports the suggestion that this is a genuine substrate/product binding site. Therefore, we refer to this site as oxidation site 1 (OS1). For visualization of the *Mv*BOx surface belonging to OS1 refer to Fig. S9.

In the structure W396A:FECN, a ferricyanide ion is bound in the same site but shifted towards the T1Cu site, with the closest observed distance to T1Cu now being 9.2 Å (Fig. S8b), and interacting through a water molecule with His398 which coordinates T1Cu. It still interacts with the main chain nitrogen atoms of Asn394 and Gly395 but now through two of its cyanide moieties. It also interacts with the main chain nitrogen atom of Ala396 and with Arg356 via two adjacent cyanide moieties (Fig. 3b).

Electron density for several other ferricyanide ions was identified and modeled in both WT:FECN and W396A:FECN (not shown) but none of them in close proximity of the T1Cu site. Some of these additional ferricyanide ions are involved in the formation of crystal contacts. A similar behavior of ferricyanide was observed in crystals of another MCO, two-domain laccase from *Streptomyces coelicolor*^44^.

### Analysis of enzymatic activity of WT and mutant *Mv*BOx

**Figure 4:**
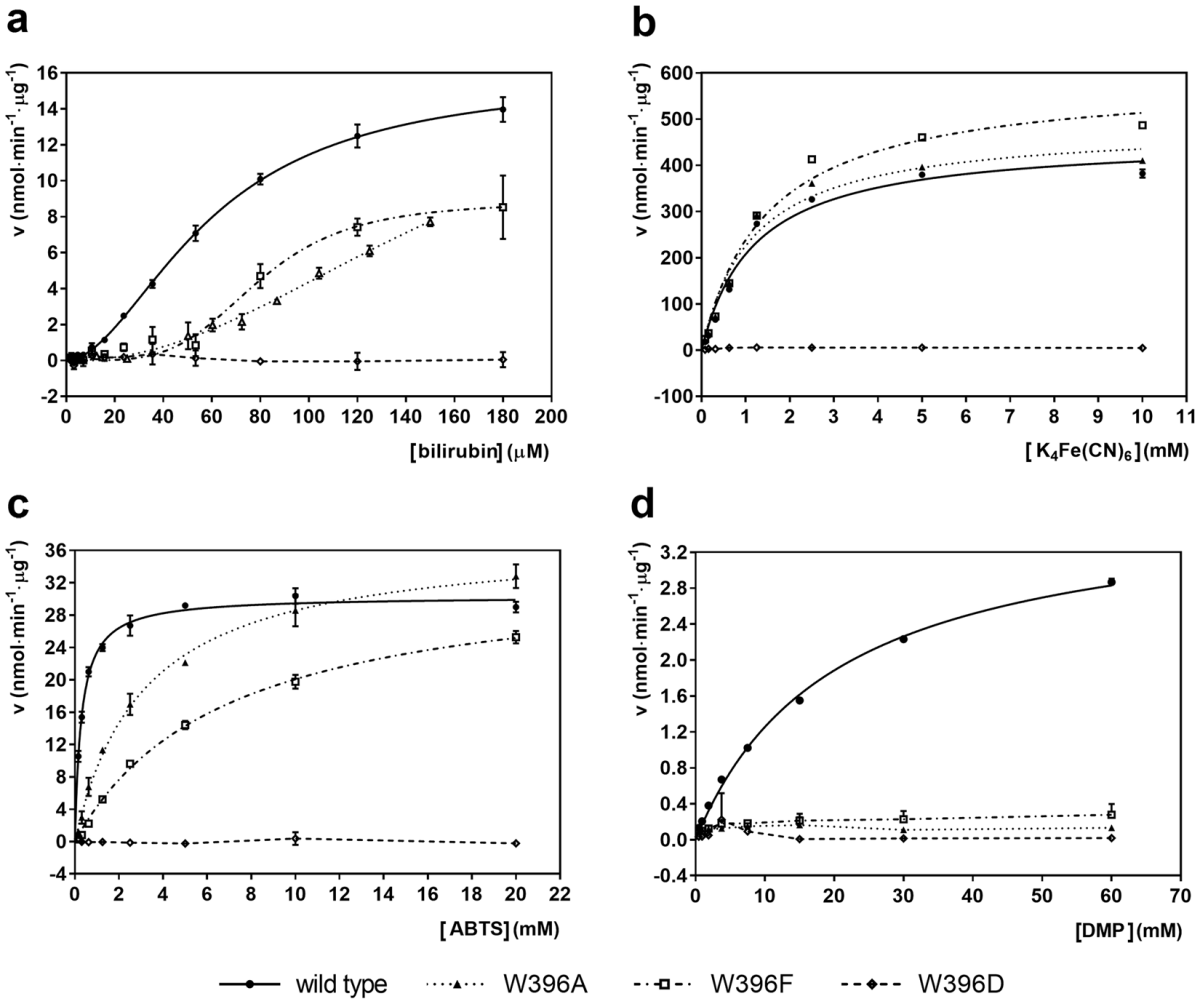
Oxidation of (**a**) bilirubin; (**b**) K_4_Fe(CN)_6_; (**c**) ABTS; and (d) DMP by MvBOxWT (solid line, ●), MvBOxW396A (dotted line, ▲), MvBOxW396F (dot-dash line, ▢), and MvBOxW396D (dashed line, ◆). Plots show the reaction velocity v as a function of substrate concentration. The curves were fitted using the program GraphPad Prism 7.02 (GraphPad Software). Standard deviations are marked as error bars. In the case of the mutant MvBOxW396D (all substrates) and all mutants (only DMP), a simple connecting line was used to link the mean values.

Enzymatic activities of *Mv*BOxWT and of the variants W396A, W396F, and W396D were analyzed using four different substrates: inorganic ([Fe(CN)_6_]^4−^ (ferrocyanide); substituted phenol 2,6-dimethoxyphenol (DMP); 2,2′-azinobis-(3-ethylbenzothiazoline-6-sulfonate (ABTS) as a standard substrate for MCOs analysis; and bilirubin as the canonical substrate for bilirubin oxidase (Fig. S1). Kinetic profiles are presented in Fig. 4 and kinetic parameters are summarized in Table 1. The kinetic profile for oxidation of bilirubin always showed sigmoidal dependence and the parameters were calculated using Eq. 1 (Materials and Methods). Kinetic parameters for oxidation of ferrocyanide, ABTS, and DMP were calculated using the Michaelis-Menten equation, providing the data converge. Otherwise the kinetic parameters remained uncalculated and a simple connecting line was used in the graphs.

**Table 1:** Kinetic parameters for oxidation of bilirubin, K_4_Fe(CN)_6_, ABTS, and DMP calculated for the measurements shown in Fig. 4. The parameters were calculated with use of the Michaelis-Menten equation (*K*_M_, *V*_max_) for K_4_Fe(CN)_6_, ABTS, and DMP. The allosteric sigmoidal equation (Equation 1, *K*_1/2_, *V*_max_, *h*) was used for bilirubin oxidation. The parameters for DMP as substrate and mutant enzymes could not be calculated due to almost zero activity.

Substrate Enzyme variant	*K*_*M*_ (mM)	*V*_max_ (nmol·min^−1^ μg^−1^)	*K*_1/2_ (mM)	*V*_max_ (nmol·min^−1^·μg^−1^)	*h*
**Bilirubin**					
*Mv*BOxWT			0.060 ± 0.002	15.8 ± 0.4	1.9 ± 0.1
W396A			0.160 ± 0.050	17.0 ± 6.0	2.2 ± 0.3
W396F			0.079 ± 0.004	8.8 ± 0.5	(4.1 ± 0.7)^#^
**K**_**4**_ **Fe(CN)**_**6**_					
*Mv*BOxWT	1.2 ± 0.2	460 ± 20			
W396A	1.2 ± 0.2	490 ± 20			
W396F	1.5 ± 0.2	590 ± 20			
**ABTS**					
*Mv*BOxWT	0.30 ± 0.02	30.3 ± 0.3			
W396A	3.1 ± 0.2	37.5 ± 0.9			
W396F	6.8 ± 0.4	33.8 ± 0.9			
**DMP**					
*Mv*BOxWT	20.1 ± 0.9	3.78 ± 0.08			

^#^The value of *h* lies within a range of 2–4. Exact value cannot be determined due to the high error present in some of the points obtained for the measurement of bilirubin oxidation by mutant W396F.

#### Oxidation rate and affinity of bilirubin are affected by mutations of Trp396

Oxidation of bilirubin was measured by detection of decrease of bilirubin concentration over time. For calculations of kinetic parameters, the values of absorbance decrease with an inverted sign were used. Since the F test and the AICc (corrected Akaike’s Information Criterion) calculations test confirmed that the measured data should be interpreted by a sigmoidal dependence on bilirubin concentration, the allosteric sigmoidal equation (Eq. 1 in Materials and Methods) was used to fit the data (Fig. 4a). The *K*_1/2_ values for the wild type and the W396F variant are comparable (0.060 ± 0.002 mM and 0.079 ± 0.004 mM, respectively) but *V*_max_ of W396F (8.8 ± 0.5 nmol·min^−1^ ·μg^−1^) is roughly a half of the wild type value (15.8 ± 0.4 nmol·min^−1^ ·μg^−1^). Kinetic parameters for the oxidation of bilirubin using the W396A variant are determined with considerably greater errors in comparison with the other *Mv*BOx variants. In this case, kinetic measurements at saturating bilirubin concentrations (>160 µM) were attempted but yielded high errors and measurements were not interpretable by mean values. The W396D variant showed practically zero activity ([−0.4 ± 2.8] % of *Mv*BOxWT reaction velocity with 120 μM bilirubin for 2 min). The results are summarized in Fig. 4a and Table 1.

#### Oxidation of ferrocyanide is significantly affected only for mutant Trp396Asp

*Mv*BOxWT, *Mv*BOxW396A, and *Mv*BOxW396F showed similar kinetic parameters for oxidation of ferrocyanide (Fig. 4b, Table 1). The mutation W396D led to a considerable decrease in the enzymatic activity ([1.27 ± 0.06] % of reaction velocity of *Mv*BOxWT with 10 mM K_4_Fe(CN)_6_ as substrate for 3 minutes).

#### Oxidation of ABTS is affected by mutations of Trp396

The affinity to the substrate ABTS significantly decreased for variants W396A and W396F (3.1 ± 0.2 mM and 6.8 ± 0.4 mM, respectively) compared to *Mv*BOxWT (0.30 ± 0.02 mM). The *V*_max_ value (30.3 ± 0.3 nmol·min^−1^·μg^−1^ for *Mv*BOxWT) slightly increased for both W396A and W396F (37.5 ± 0.9 nmol·min^−1^·μg^−1^ and 33.8 ± 0.9 nmol·min^−1^·μg^−1^, respectively). The mutation W396D led to null activity ([−0.8 ± 0.3] % of *Mv*BOxWT reaction velocity for 20 mM ABTS as substrate for 3 min). The results are summarized in Fig. 4c and Table 1.

#### All mutations of Trp396 diminish oxidation of DMP

All mutants show negligible activity compared to *Mv*BOxWT. Therefore, a simple connecting line was used to connect the mean values in the kinetic profile (Fig. 4d). The variants W396A, W396F, and W396D reached 5.0 ± 2.0%, 10.0 ± 3.0%, and 0.6 ± 0.3% of the *Mv*BOxWT reaction velocity for 30 mM DMP as substrate in 4 min, respectively. The results are summarized in Fig. 4d and Table 1.

## Discussion

### Structure of *Mv*BOx is maintained under extreme pH

The comparison of the *Mv*BOx structures from acidic (pH 3.1, PDB ID 6I3J and pH 5.0, PDB ID 6IQZ) and basic (pH 8.7, PDB ID 2XLL) crystallization conditions proves that the *Mv*BOx structure did not change with the change of pH (Fig. S2). It should be noted that the same glycosylation pattern (at Asn472 and Asn482) is present under all the studied pH values and that the position of glycosylation differs from fungal laccases^45^. This lack of pH-dependent structural differences suggests that different pH optima for the individual types of substrates cannot be connected with structural changes (e.g. pH 4 for oxidation of ABTS;^6^ pH around 8.4 for oxidation of bilirubin^18^). This observation is in agreement with the findings of Otsuka *et al*., according to which the pH optimum of *Mv*BOx is predominantly determined by the difference between the redox potentials of *Mv*BOx and of the particular substrate^6^.

### Trp396–His398 covalent crosslink is natively present in *Mv*BOx and its existence is not pH dependent

As the existence of the Trp396–His398 covalent crosslink in *Mv*BOx was confirmed using LC–MS/MS (Fig. S3) we can conclude that it is present in the enzyme in solution. Moreover, it is present not only in the structure from the strongly acidic crystallization condition reported here (PDB ID 6I3J), but also in the previously reported structures of *Mv*BOx from acidic (6IQZ) and basic condition (2XLL), as discussed by Akter *et al*.^40^. Based on these results it is safe to conclude that the Trp–His adduct is present in native *Mv*BOx in solution over a broad range of functionally relevant pH values. This is important for relevance of the mutagenesis–activity studies presented in this work.

Trp396 and His398, along with several residues in their proximity, are conserved in many homologues of *Mv*BOx from fungi and bacteria (Fig. S10). Conservation of these residues indicates the possible existence of the same Trp–His crosslink in bilirubin oxidases or closely related enzymes from other organisms.

### Trp396–His398 adduct modifies coordination of T1Cu

The presence of the Trp396–His398 adduct in *Mv*BOx has measurable effects on the coordination of T1 copper as described in the Results section. The significant tilt of the His398 imidazole moiety coordinating T1Cu is also observable in electron density in the structure of *Mv*BOx wild type 2XLL^8^ and present in the structure of *Mv*BOx wild type determined by Akter *et al*. (PDB ID 6IQZ^40^). However, this tilt is not in the structure of *Mv*BOx M467Q which, due to the mutation, does not contain the Trp396–His398 crosslink (PDB ID 6IQY^40^). As the T1Cu site in the structures W396A:FECN (PDB ID 6I3K) and W396F (6I3L) did not show this tilt either, we conclude that it is caused by the coordination of T1Cu by the Trp396–His398 adduct (Fig. 2).

The longer coordination distance of T1Cu to His462-N^δ1^ observed in the case of WT:FECN (Fig. 2) is also present in the structure 2XLL (refined to approximately 2.2 Å^8^), but not in the structure of *Mv*BOx wild type determined by Akter *et al*.^40^. And so this change of T1Cu coordination distance in connection with the adduct presence requires further investigation.

### Trp396–His398 crosslink formation

To the best of our knowledge, the Trp–His crosslink has been observed only in *Mv*BOx, but covalent crosslinks between side chains of residues Tyr and Cys, Tyr and Met, Tyr and Trp, Tyr and His, and Cys and His were identified in several enzymes, with three types of copper-containing oxidases among them^46^. Tryptophan side chain, especially the indole group, is reactive and susceptible to chemical modifications^47^. It can undergo electrochemical oxidation on carbon C^δ1^ ^48^ with an oxidation peak potential of 0.64 V at pH 7^49^. The redox potential of the T1Cu site was identified as the main reason for the Trp396–His398 adduct formation in *Mv*BOx also by Akter *et al*. in their recent work^40^.

According to our analysis, the observed rotamer of the Trp396 side chain is the only standard tryptophan rotamer acceptable for this site without any clashes with the surrounding residues. This is true for both, the Trp–His adduct (PDB ID 6I3J, this work) and also for the structure without the crosslink between Trp396 and His398 (6IQY^40^). Therefore, we can conclude that the particular fold of the enzyme in this region is actually in favor of the adduct formation by placing the indole group in a close contact with the His398 side chain.

### Ferricyanide binds to positively charged site near Trp396–His398 adduct

The surface of *Mv*BOx near the T1Cu site is positively charged (Fig. S11a), as observed previously in investigations of the orientation of *Mv*BOx molecules on negatively charged electrodes for direct electron transfer^50–52^. OS1, the ferricyanide binding site of *Mv*BOx (Fig. 3a), is a part of this positively charged surface near the T1Cu site. Thus, this crystallographically identified site OS1 is the central site for substrate oxidation of *Mv*BOx substrates and its properties must influence substrate binding and catalytic efficiency of the enzyme.

### Trp396–His398 adduct has no significant structural role in *Mv*BOx

The structure of *Mv*BOx was not affected by the mutation of Trp396 to Ala and Phe (Figs S6 and S7). An X-ray structure of *Mv*BOxW396D could not be determined, because this variant did not crystallize. Nevertheless, based on the CD (Fig. S4), and UV-VIS (Fig. S5) spectra it can be concluded that also this mutation did not change the secondary structure composition of *Mv*BOx and the existence of the T1Cu site. So neither the elimination of the Trp396–His398 adduct, nor functional changes of the T1Cu site environment (direct solvent access to His398, aromatic residue without the crosslink or introduction of a negatively charged residue) have significant effects on the *Mv*BOx structure. The presence of the Trp396–His398 adduct in *Mv*BOx plays a minor role in its thermal stability (Fig. S12) as follows from the slight decrease of the melting temperature (*T*_m_) of the W396A and W396F mutants (by about 5 °C and 7 °C, respectively). The W396D mutation caused a significant decrease of *T*_m_ (by about 20 °C). This can be explained by exchanging a hydrophobic residue (Trp) for a hydrophilic one (Asp) and also possibly by introducing negative charge to the otherwise positively charged site (Fig. S11b).

### Trp396–His398 adduct participates in substrate binding and oxidation, depending on substrate type

The kinetic data measured for *Mv*BOxWT and the Trp396 mutants (Table 1, Fig. 4) clearly show that different substrates utilize the Trp396–His398 adduct in different ways and that the adduct (or possibly Trp396) is crucial only in the case of DMP as substrate. Simultaneously, mutation of Trp396 to Asp disabled oxidation of all substrates as this mutation changed the electrostatic potential distribution in OS1 (Fig. S11b).

#### Trp396–His398 adduct is important in bilirubin oxidation

Our kinetic data for bilirubin show that the Trp396–His398 adduct is most probably involved in both, the substrate binding and its oxidation. All the investigated mutations always led to a significant decrease of the catalytic efficiency (*V*_max_/*K*_1/2_) for bilirubin. The W396A mutant binds bilirubin with a lower affinity when compared to the wild type. The W396F mutant retained affinity comparable with that of the wild type, but with a lower maximal reaction velocity. In addition, both mutants showed much more distinctive allosteric effect (Table 1, Fig. 4a). On the structural level, the mutation of Trp396 to Phe allows for conservation of the aromatic moiety in the proximity of the T1Cu site (Fig. 2c), whereas the mutation to Ala disposes of the aromatic moiety and instead allows for solvent access to His398 coordinating T1Cu (Fig. 3b). Considering these differences (both structural and in the enzymatic activity) it is clear that the Trp396–His398 adduct is involved in binding of bilirubin, although bilirubin can still bind to *Mv*BOx mutants not containing tryptophan at the position 396. One of the possible explanations for the substantial decrease in bilirubin oxidation observed in the M467Q mutant^5^, which contains Trp396 but without the crosslink to His398^40^, is the usage of low substrate concentration (27 μM). At this concentration, the activity may be influenced by the allosteric effect observed in the mutants W396A and W396F (Fig. 4a).

#### Trp396–His398 adduct and ferrocyanide oxidation

In the case of ferrocyanide, the kinetic parameters of *Mv*BOxWT and both mutants W396A and W396F are similar. Ferricyanide (product) binds in the same site in both *Mv*BOxWT and *Mv*BOxW396A, although in two different poses. And even if Trp396 forms a part of the ferricyanide binding site in the wild type, it is not necessary for its binding. The main structural features, which ferricyanide utilizes, are the main chain nitrogen atoms of Asn394 and Gly395 together with the side chain of Arg356 (Fig. 3). The main difference in binding of ferricyanide between *Mv*BOxWT and *Mv*BOxW396A lies in the fact that the replacement of Trp by Ala allows ferricyanide to bind closer to T1Cu. Ferricyanide in the W396A mutant interacts with T1Cu-coordinating His398 through a water molecule, possibly mediating electron transfer. The W396D mutant is almost inactive (~1% of the wild type activity). Possibly, the negatively charged aspartic acid side chain either compensates the partial positive charges of the Asn394/Gly395 main chain nitrogen atoms or directly repels the negatively charged ferrocyanide ion and so interferes with its binding. Preservation of ferrocyanide activity was also reported for the M467Q mutant^5^. From these results, it can be concluded that the Trp396–His398 adduct itself is not important for binding of ferrocyanide. The geometry and positive electrostatic potential of OS1 are sufficient for its binding close enough in the proximity of T1Cu for efficient electron transfer (within 14 Å limit^43^; see Fig. S8). Although the adduct is probably involved in electron transfer in *Mv*BOxWT, its presence is not crucial in the case of ferrocyanide oxidation as electron transfer can be realized using another path, possibly *via* the protein main chain of the loop forming OS1 (residues 394–398) or *via* water molecule in the case of W396A:FECN.

#### Trp396–His398 adduct and ABTS oxidation

The mutations of Trp396 to Ala or Phe have similar effects. ABTS binds to both mutants with a significantly lower affinity, but the maximal velocity is not affected. So, in the case of ABTS, the Trp396–His398 adduct plays a role in the substrate binding. The electron transfer in this case can be realized *via* the adduct or *via* another path (possibly main chain of the loop forming OS1).

#### Trp396–His398 adduct and DMP oxidation

As all the mutants studied here had a significantly lower activity toward DMP when compared to the wild type of *Mv*BOx, the kinetic parameters could not be calculated. Attempts to obtain structural information for a complex of *Mv*BOx and DMP failed. It remains to be deciphered if the observed lack of activity towards DMP in the mutants is caused by a lower substrate affinity, disturbance of the electron transfer path upon elimination of the Trp396–His398 adduct or simply by the unfavorable difference in the redox potential between DMP and the individual enzyme variants.

### OS1, Trp–His adduct, and electron transfer

Based on the presented results, we propose that all the substrates studied here utilize the oxidation site 1 (OS1), although the different substrates very likely bind at or near OS1 in a different way. OS1 comprises of residues which contribute only toward substrate binding (Asn197, Arg356, and Asn394) and residues which also participate in electron transfer from substrate to T1Cu (Trp396 of the Trp–His adduct, Gly395, and possibly also Asn394). In the case of ferrocyanide oxidation, the structures of the complexes enable a deeper analysis of the mechanism of substrate binding and oxidation, including the details of ferro/ferricyanide interactions with *Mv*BOx.

For all substrate types, it seems that oxidation always relies, besides the difference between the redox potentials of substrate and *Mv*BOx, on the interplay between the Trp396–His398 adduct and the main chain nitrogen atoms of the 393–395 loop forming the positively charged binding site. Other residues in the vicinity of the Trp396–His398 adduct may also play roles in substrate binding and/or oxidation (most likely including Arg356, Trp361, and Asn197). The Trp–His adduct is not the only possible electron transfer route in *Mv*BOx. The whole loop 393–396 is important in substrate binding, adduct formation, and electron transfer. For some substrates, it likely participates in electron transfer *via* its main chain atoms.

## Conclusion

Formation of the covalent link between the side chains of Trp396 and T1 copper-coordinating His398, confirmed in *M*. *verrucaria* bilirubin oxidase, is facilitated by the enzyme fold and local organization of the protein chain. The Trp396 indole ring effectively mimics the position of substrates in other multicopper oxidases and is activated by the T1Cu site redox potential. The adduct participates in formation of the oxidation site 1 involved in substrate binding and oxidation of all substrates including larger and/or aromatic compounds and bilirubin. Mutations of Trp396 influence the enzyme activity but not the enzyme structure (except the replaced residue). Based on the mutagenesis and kinetics results, different substrate types must bind in the proximity of the Trp–His adduct, while at the same time utilize this unique substrate oxidation site differently. As most of the studied substrates, including bilirubin, are oxidized even in the absence of this adduct, its role in electron transfer is not crucial. In the case of ferricyanide binding, except the Trp–His adduct, also Arg356 and the loop 393–398 are important for substrate/product-enzyme interactions.

## Materials and Methods

### Cloning of bilirubin oxidase wild type

Gene for bilirubin oxidase was amplified from *M*. *verrucaria* (*A*. *verrucaria*) strain ATCC24571 by primers 5′-AGAGCGAUACCATGTTCAAACACACAC and 5′-AACGTCACGUCTACTCGTCAGCTGCGGC having overhangs that incorporated a single deoxyuracil residue (dU) flanking the 3′ end of the homology region. The amplified DNA (band of 2059 base pairs) was used for USER^®^ cloning into an expression vector.

### Construction of mutated variants of bilirubin oxidase

Genes of all variants were generated by spliced overlap extension (SOE) polymerase chain reaction (PCR) with flanking primers 5′-AGAGCGAUACCATGTTCAAACACACAC (forward) and 5′-AACGTCACGUCTACTCGTCAGCTGCGGC (reverse) and hybrid primers containing the desired codon change. The resulting oxidase variant genes were cloned into an expression vector by USER^®^.

### Expression and purification

All samples were expressed and purified similarly as described in Kovaľ *et al*.^53^. In detail, constructs were verified by DNA sequencing and transformed into protoplasts of *Aspergillus oryzae* for expression driven by the TAKA amylase promoter. The transformed strain of *A*. *oryzae* was grown for 3 days at 30 °C and 200 rpm in shake flasks containing MDU-2BP (45 g of maltose, 1 g of MgSO_4_.7H_2_O, 1 g of NaCl, 2 g of K_2_SO_4_, 12 g of KH_2_PO_4_, 7 g of yeast extract, 0.5 ml of trace elements, and 1% (w/v) urea per l). Additional CuSO_4_ was added to the shake flasks to a final concentration of 0.5 mM. The fermentation broth was sterile filtered to remove fungal hyphae. Salts and other low molecular weight solutes were removed by ultrafiltration. 1 M Tris/HCl, pH 7.5 was added to the resulting retentate to a final concentration of 25 mM. pH and ionic strength were determined to be within the acceptable range for anion exchange chromatography. The chromatography was then conducted with an ÄKTA Prime instrument (Amersham Biosciences). Briefly, the protein was bound to a column with 20 ml Q Sepharose High Performance pre-equilibrated with 25 mM Tris/HCl, pH 7.5. After a thorough wash with the equilibration buffer, the bound protein was eluted from the column with a linear NaCl gradient (0–0.5 M) in the equilibration buffer over ten column volumes. *Mv*BOx eluted at approximately 250 mM NaCl. Collected fractions containing pure *Mv*BOx, as estimated by SDS-PAGE, were pooled. All purification steps were carried out at room temperature.

### Mass spectrometry

*Mv*BOxWT was digested by trypsin. Peptides were further analyzed by LC-MS/MS using a 15 T solariX FT-ICR mass spectrometer (Bruker Daltonics) operating in positive mode.

### *Mv*BOx activity assay

The steady-state kinetic parameters for all *Mv*BOx variants were determined using four distinct substrates: potassium ferrocyanide (further referred to as ferrocyanide because only [Fe(CN)_6_]^4−^ anion undergoes oxidation), 2,2′-azino-bis(3-ethylbenzothiazoline-6-sulphonic acid) (ABTS), bilirubin, and 2,6-dimethoxyphenol (DMP). All reactions were monitored spectrophotometrically using a CLARIOstar Monochromator Microplate Reader (BMG Labtech, Ortenberg, GE). Reactions were monitored for substrates [Fe(CN)_6_]^4−^ and ABTS at 420 nm (ε_420_ = 1.04 mM^−1^ cm^−1^ ^54^ and ε_420_ = 36 mM^−1^ cm^−1^ ^55^, respectively), for DMP at 468 nm (ε_468_ = 14.8 mM^−1^ cm^−1^)^56^, and for bilirubin at 440 nm (ε_440_ = 56.3 mM^−1^ cm^−1^)^57^. Single reactions (100 μl total volume) were prepared in triplicates and run in black 96-well plates (BRAND, Wertheim, GE). All reactions were carried out at 27 °C.

Oxidation of K_4_Fe(CN)_6_ (0.1–10 mM) was done using 50 mM Bis-Tris, pH 6 and 25 mM NaCl with 0.15 μg of enzyme for 3 min (total volume 100 μl). Oxidation of ABTS (0.2–20 mM) was done using 100 mM sodium acetate, pH 4 with 0.06 μg of enzyme for 3 min. Oxidation of bilirubin (1–180 μM) was done using 200 mM Tris-HCl, pH 8.7 with 0.02 μg of enzyme for 3 min. Oxidation of DMP (0.5–60 mM) was done using 50 mM Bis-Tris, pH 6.8 and 25 mM NaCl with 0.4 μg of enzyme for 4 min.

Steady-state kinetic parameters (maximal velocity *V*_max_ and Michaelis-Menten constant *K*_M_) were calculated using the Michaelis-Menten non-linear regression equation with GraphPad Prism version 7.02 for Windows (GraphPad Software, La Jolla California USA, www.graphpad.com). In the case of bilirubin, the non-hyperbolic data with “S-shaped” sigmoidal behavior were taken into consideration by application of the Hill equation (Eq. 1, according to GraphPad Prism 7.02 Software):

1 $$v=\frac{{V}_{max}\cdot {[S]}^{h}}{{{K}_{1/2}}^{h}+{[S]}^{h}}$$

*K*_1/2_ is the concentration of substrate at the half of the maximal velocity *V*_max_, *h* is a Hill slope. When *h* = 1, *K*_*1/2*_ is the *K*_M_ value. To evaluate the best fit, the F test and the corrected Akaike’s Information Criterion (AICc) calculations were performed using the GraphPad QuickCalcs Web (http://www.graphpad.com/quickcalcs/ConfInterval1.cfm, accessed May 2017). The equation best fitting the particular data was used for the final calculation of the kinetic parameters.

### Crystallization, data collection, structure solution, and refinement

For crystallization, *Mv*BOxWT, *Mv*BOxW396A, and *Mv*BOxW396F were concentrated in the storage buffer (25 mM Tris/HCl, pH 7.5 with 250 mM NaCl) to 25 mg ml^−1^ using a 10 kDa cut–off Nanosep centrifugal device (Pall Corporation). Initial screening for crystallization conditions was done only for *Mv*BOxWT using the hanging drop vapor diffusion setup with a protein to reservoir drop volume ratio of 1:1 (0.5 μl + 0.5 μl). Screening using several commercially available crystallization screens did not yield hits. As the theoretical pI of mature *Mv*BOx is 4.97, a limited crystallization screen containing acidic conditions was used^58^. Initial hits were observed after 1–3 days using 25% (w/v) PEG 3350, 0.1 M citric acid, pH 3 as a reservoir solution and this condition was further optimized. Crystal of *Mv*BOxWT used for X-ray analysis was obtained using the hanging drop vapor diffusion method. The drop was composed of 1 μl of protein mixed with 1 μl of reservoir solution (0.1 M succinic acid, 14% (w/v) polyethylene glycol 3350). Succinic acid was not titrated. Solution obtained by mixing the reservoir solution with the storage buffer in ratio 1:1 had pH 3.1. Crystals of *Mv*BOxW396A and *Mv*BOxW396F were obtained using the same reservoir solution and sitting drop setup with 0.5 μl + 0.5 μl drop volume. All crystallization trials were done at 18 °C.

Prior to vitrification using liquid nitrogen, crystals of *Mv*BOxWT and *Mv*BOxW396A were soaked in reservoir solution containing 10 mM K_4_Fe(CN)_6_ and cryoprotectant for 90 seconds (for *Mv*BOxWT, a combination of 15% (w/v) polyethylene glycol 200, 1% (v/v) glycerol, 1% (v/v) ethylene glycol, and 1% (v/v) propylene glycol; for *Mv*BOxW396A 25% (v/v) glycerol). Both crystals changed appearance from blue to transparent indicating reduction of T1Cu (Supplementary video sequences 1 and 2). Soaking solution changed color from pale yellow to darker yellow indicating oxidation of ferrocyanide ([Fe(CN)_6_]^4−^) to ferricyanide ([Fe(CN)_6_]^3−^). An *Mv*BOxW396F crystal was soaked in solution containing 25% (v/v) glycerol and 10 mM pyrogallol in reservoir solution for 60 seconds. An *Mv*BOxWT crystal was mounted in nylon CryoLoop (Hampton Research), *Mv*BOxW396A and *Mv*BOxW396F crystals in round LithoLoop (Molecular Dimensions). For WT and W396A crystals, diffraction data were collected with 0.91841 Å wavelength at beamline BL 14.1 of the BESSY II synchrotron radiation source (Helmholtz Zentrum Berlin, DE) at 100 K. Data for WT:FECN were collected using a MAR Mosaic CCD 225 detector and a mini kappa goniometer, data for W396A:FECN were collected using a Dectris Pilatus 6 M detector and a mini kappa goniometer^59^. Data for W396F were collected at 100 K and with 1.3418 Å wavelength (gallium K_α_) using a D8 VENTURE diffractometer, a Photon II detector (Bruker) and a METALJET X-ray source (Excillum). Data were processed and scaled using XDS or XDSGui^60^ and merged using Aimless^61^. For all structures, the phase problem was solved by molecular replacement using Molrep^62^ and the structure of *Myrothecium verrucaria* bilirubin oxidase (PDB code 2XLL)^8^ as a template.

All three structures were manually edited using Coot^63^ and refined using restrained refinement in REFMAC5^64^ with *R*_free_ as a cross validation method. The last refinement cycle was done using all reflections. Structures were validated using the tools implemented in Coot, the structure-validation web service Molprobity^65^ and the wwPDB Validation service^66^. Data collection and processing statistics are reported in Table 2.

**Table 2:** Statistics of data collection and processing and structure refinement parameters for *Mv*BOx and its variants. Values in parentheses are for the highest resolution shell.

Structure	Wild type + ferricyanide	W396A mutant + ferricyanide	W396F mutant
PDB ID	6I3J	6I3K	6I3L
**Data collection**			
X-ray source	BESSY II, BL14.1	BESSY II, BL14.1	MetalJet D2
Wavelength (Å)	0.91841	0.91841	1.3418
Detector	MAR mosaic CCD	Pilatus 6 M	Photon II
Detector distance (mm)	313.5	266.7	75
No. of oscillation images	217	1000	400
Exposure time per image (s)	2	0.2	90
Oscillation width (°)	0.5	0.1	0.3
Space group	*F*222	*F*222	*F*222
Unit-cell parameters *a*, *b*, *c* (Å)	134.4, 203.9, 226.7	136.9, 201.8, 217.9	136.3, 200.7, 217.1
Resolution range (Å)	47.30–2.59 (2.67–2.59)	47.93–1.60 (1.63–1.60)	45.54–2.10 (2.14–2.10)
No. of observations	313528 (21715)	739043 (37457)	400760 (13579)
No. of unique reflections	48305 (4411)	195423 (9686)	83782 (3866)
Data completeness (%)	100.0 (99.9)	99.6 (99.8)	97.3 (82.4)
Redundancy	6.5 (4.9)	3.8 (3.9)	4.8 (3.5)
Mosaicity (°)	0.22	0.07	0.13
Average *I/σ(I)*	13.5 (2.0)	9.2 (1.5)	7.3 (2.1)
Solvent content (%)	62.2	61.0	60.4
*R*_merge_	0.144 (0.858)	0.074 (0.635)	0.188 (0.649)
*R*_meas_	0.157 (0.963)	0.086 (0.738)	0.212 (0.759)
*CC*(1/2)	0.994 (0.608)	0.998 (0.700)	0.990 (0.697)
Wilson *B* factor (Å^2^)	30.7	10.9	16.7
**Refinement**			
*R*_work_	0.160	0.131	0.159
*R*_free_	0.226	0.154	0.196
Average *B* factor (Å^2^)	36.1	15.5	19.8
R.m.s.d. bonds from ideal (Å)	0.009	0.011	0.009
R.m.s.d. angles from ideal (°)	1.350	1.670	1.539
Ramachandran favoured (%)	94.45	95.77	95.30
Ramachandran outliers (%)	0	0	0

The complexes of *Mv*BOxWT and *Mv*BOxW396A with ferricyanide ([Fe(CN)_6_]^3−^, product) reported here (PDB ID 6I3J and 6I3K, respectively) were obtained by soaking of the crystals in excess of ferrocyanide ([Fe(CN)_6_]^4−^, substrate). The oxidation of ferrocyanide and reduction of *Mv*BOx during the soaking of the crystal was clearly observable (see above). However, due to the fact that the trinuclear copper cluster in WT:FECN is fully reduced (Fig. S13), the observed ligand could still be ferrocyanide. Unfortunately, ferrocyanide and ferricyanide are not distinguishable at the obtained resolutions. We chose to model product (ferricyanide, [Fe(CN)_6_]^3−^) in both structures. The geometrical restraint library for the link between Trp396 and His398 was built based on data found in the crystallographic database of organic compounds (Cambridge Structural Database, The Cambridge Crystallographic Data Centre). The particular geometry of the link was identified in 3 records of CSD with ID codes CIMGUC (occurrence 4×), CIMHAJ (1×) and SEPXOC (1×). Records CIMGUC and CIMHAJ were chosen for extraction of averaged geometrical parameters, which were directly used for construction of the restraints. Orientation of the new C-N bond was restrained independently in the planes of the two corresponding planar side chains.

## Supplementary information

Supplementary video sequences 1

Supplementary video sequences 2

Supplementary information

## Data Availability

The crystal structures and corresponding data were deposited in the Protein Data Bank under the codes 6I3J (wild type in complex with ferricyanide) 6I3K (mutant W396A with ferricyanide) and 6I3L (mutant W396F). All other source data are available upon reasonable request.
