# Supplementary material for: Trp–His covalent adduct in bilirubin oxidase is crucial for effective bilirubin binding but has a minor role in electron transfer
Source: Sci Rep. 2019 Sep 23;9:13700. doi: 10.1038/s41598-019-50105-3 (PMC6757100; doi:10.1038/s41598-019-50105-3)
Supplement: Supplementary file 3 — Supplementary information [file 41598_2019_50105_MOESM3_ESM.pdf]

Supporting information

Trp–His covalent adduct in bilirubin oxidase is crucial for effective bilirubin binding but has a minor role in electron transfer

Tomáš Koval'*#[1], Leona Švecová#[1, 2], Lars H. Østergaard[3], Tereza Skálová[1], Jarmila Dušková[1], Jindřich Hašek[1], Petr Kolenko[1, 2], Karla Fejfarová[1], Jan Stránský[1], Mária Trundová[1], and Jan Dohnálek*[1]

[1]Institute of Biotechnology of the Czech Academy of Sciences v.v.i., Průmyslová 595, 252 50 Vestec, Czech Republic
[2]Faculty of Nuclear Sciences and Physical Engineering, Czech Technical University in Prague, Břehová 7, 115 19, Praha 1, Czech Republic
[3]Novozymes A/S, Krogshoejvej 36, 2880 Bagsvaerd, Denmark

*To whom correspondence should be addressed. Email: tomas.koval@ibt.cas.cz, jan.dohnalek@ibt.cas.cz; Tel: +420 325 873 758;

#These authors contributed equally.

Supplementary video sequence 1. Single crystal of *Mv*BOxWT in mother liquor before and after addition of $Fe[(CN)_6]^{4-}$ at concentration equivalent to preparation of crystals before diffraction analysis. The individual images of the sequence were acquired with approximately 2 s intervals. Crystals were vitrified at point corresponding to the length of the video sequence.

Supplementary video sequence 2. Single crystal of *Mv*BOxW396A in mother liquor before and after addition of $Fe[(CN)_6]^{4-}$ at concentration equivalent to preparation of crystals before diffraction analysis. The individual images of the sequence were acquired with approximately 2 s intervals. Crystals were vitrified at point corresponding to the length of the video sequence.

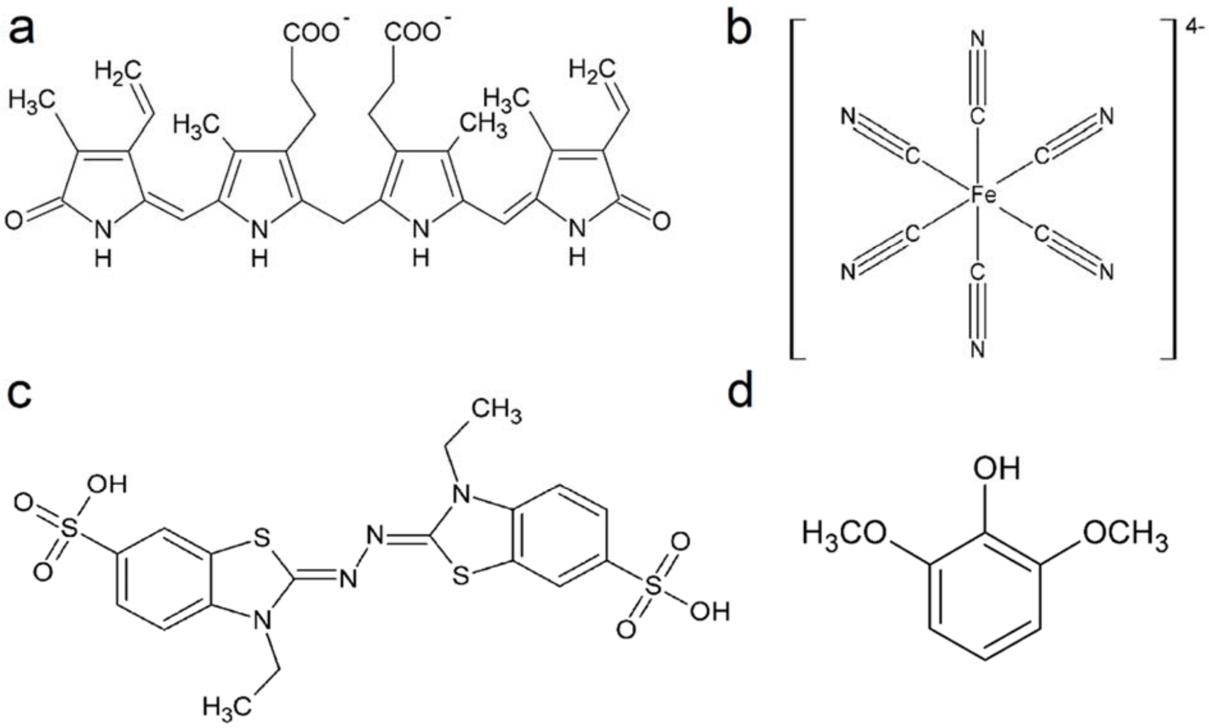

Figure S1. Structural formula of bilirubin oxidase substrates used in this study. (a) Bilirubin, **(b)** ferrocyanide ion, **(c)** ABTS (2,2'-azino-bis(3-ethylbenzothiazoline-6-sulfonic acid)), **(d)** DMP (2,6-dimethoxyphenol).

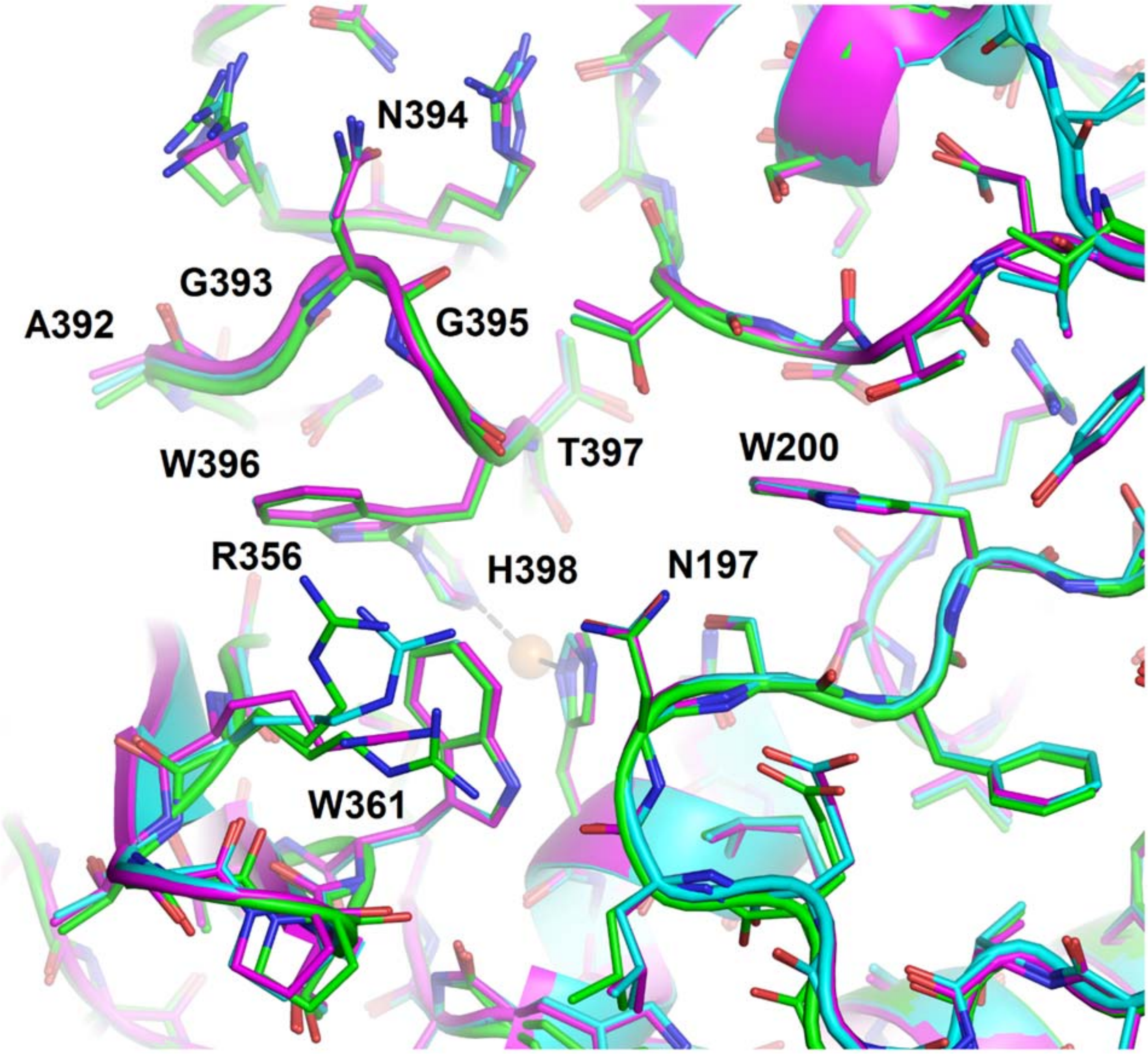

Figure S2. Superposition of WT:FECN (6I3J) with structures from acidic, and basic crystallization conditions. Spatial positions of all residues around the T1Cu site are basically the same. The structure 6I3J from the strongly acidic condition (reported here) has carbon colored green, 2XLL (1) from the basic condition magenta, and 6IQZ (2) from the acidic condition light blue. The T1Cu copper ion is shown as orange sphere. Selected residues are labeled. Molecular graphics was created using PyMOL (Schrödinger, LLC).

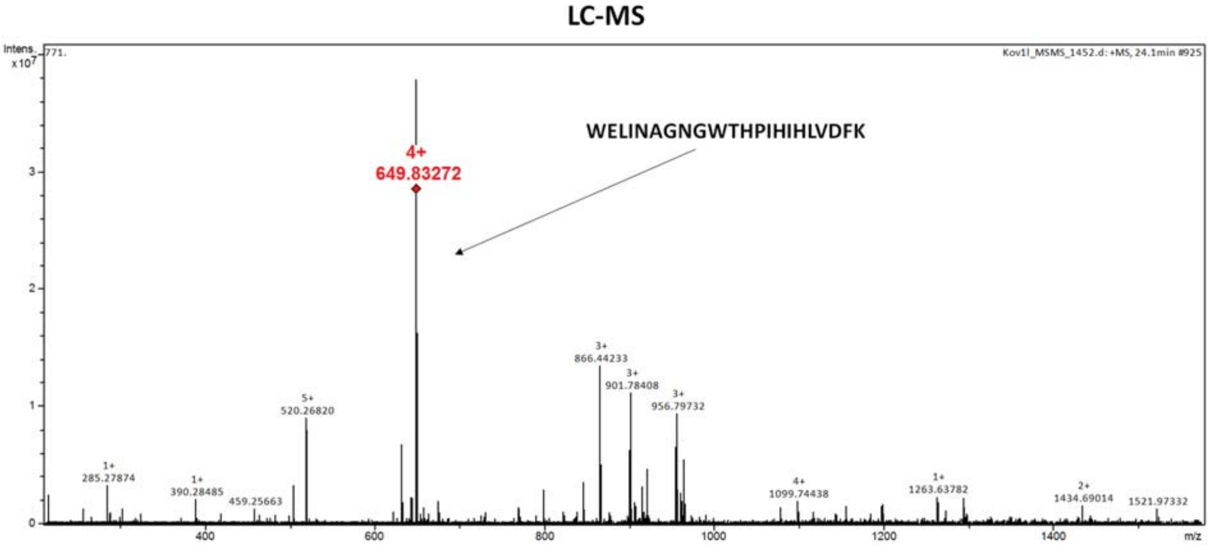

Figure S3. Liquid chromatography–mass spectrometry spectrum of peptidic fragments of *Mv*BOxWT. *Mv*BOxWT was digested by trypsin. Peptides were further analyzed by LC-MS/MS using a 15T solariX FT-ICR mass spectrometer (Bruker Daltonics) operating in positive mode. The existence of the Trp396–His398 crosslink was confirmed by the observation of ion at m/z 649.8327 (4+) corresponding to the modified WELINAGNGWTHPIHIHLVDFK peptide.

.

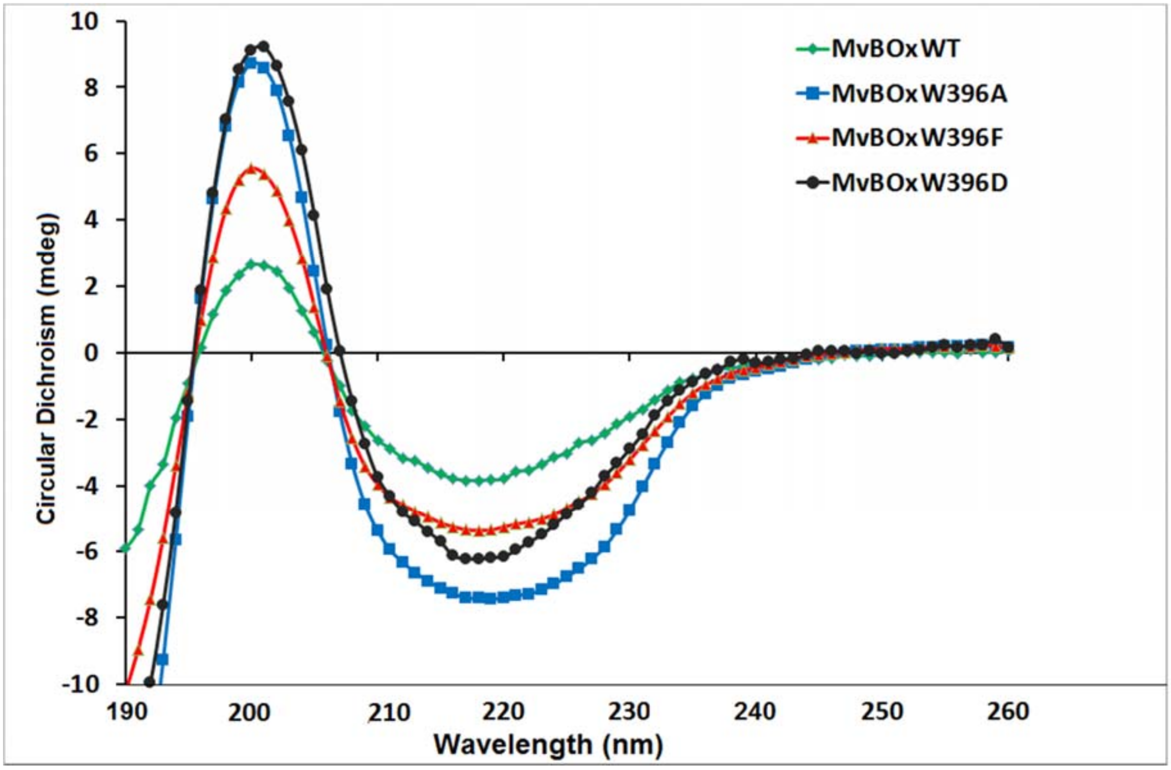

Figure S4. Circular dichroism spectra of *Mv*BOxWT and all the studied mutants. The mutation of Trp396 to alanine, phenylalanine or aspartic acid had no effect on *Mv*BOx secondary structure. Circular dichroism spectra for all samples were recorded using a 0.1 cm quartz cell and a Chirascan™–plus spectrometer (Applied Photophysics). Spectra were recorded in the range of wavelengths of 190–260 nm with a step of 1 nm at room temperature. Concentration of samples was approximately 0.2 mg ml^{-1}. Samples were diluted from stock solution by water. The resulting spectra were buffer-subtracted. The CD signal was expressed as ellipticity. The height of peaks reflects only varied concentration of the samples.

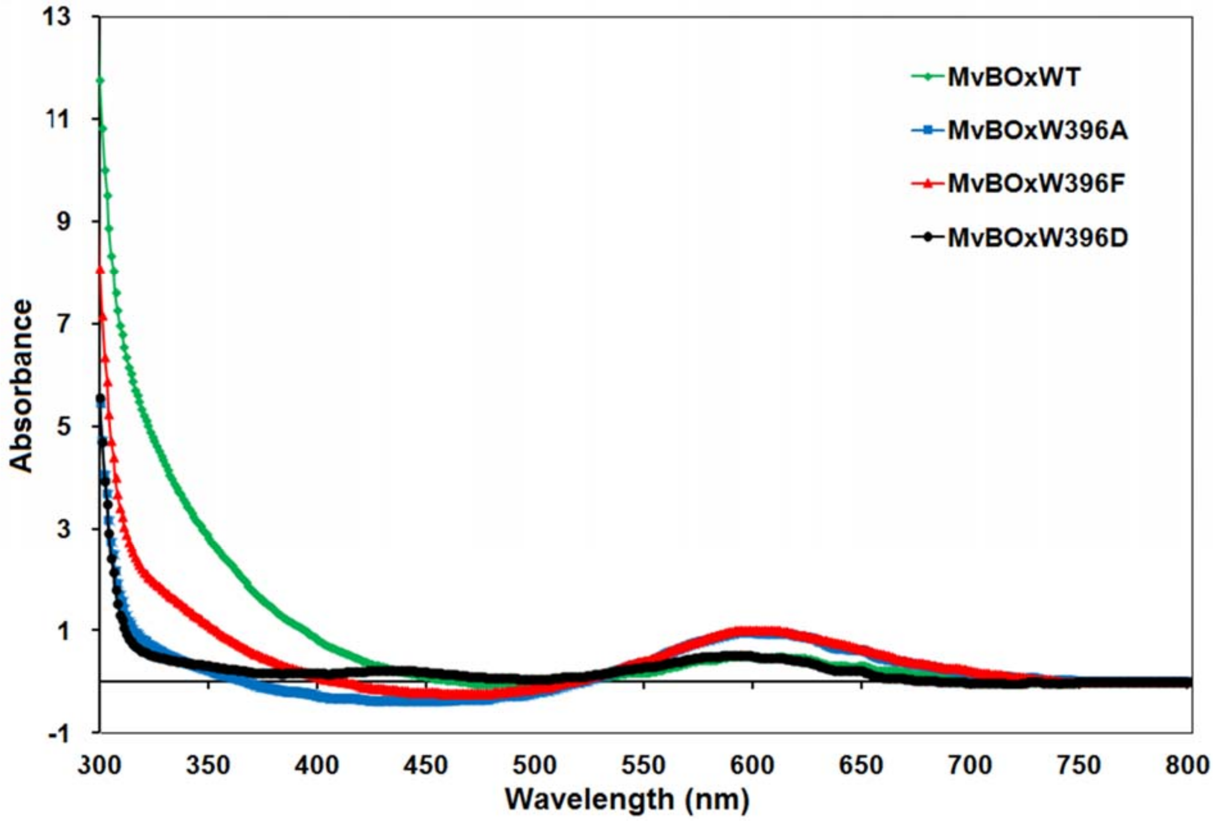

Figure S5. UV-VIS spectra of *Mv*BOxWT and of the mutants W396A, W396F, and W396D. All samples show an absorbance peak around 600 nm, typical for multicopper oxidases with oxidized T1Cu site. UV-VIS absorption spectra were collected using a DeNovix DS-11 microvolume spectrophotometer and 1.2 µl of each sample. Spectrum of buffer (25 mM Tris/HCl, pH 7.5 with 250 mM NaCl) was subtracted in all cases.

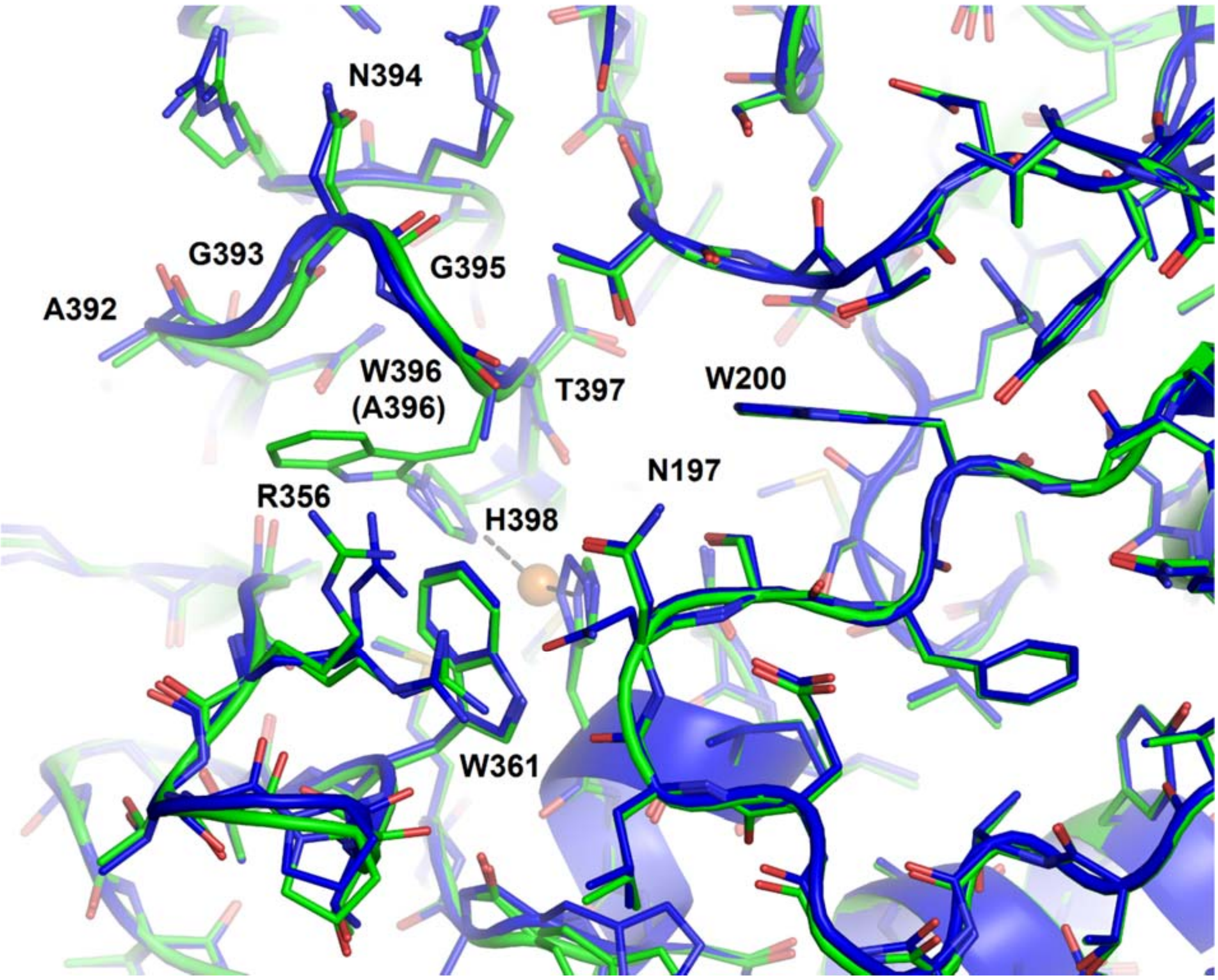

Figure S6. Superposition of the structures *Mv*BOxWT and *Mv*BOxW396A. The mutation of Trp396 to alanine did not affect the structure of the active site besides the intended lack of the indole moiety and of the crosslink (A396). The structure 6I3J of *Mv*BOxWT has carbon colored green, the structure 6I3K of *Mv*BOxW396A blue. The T1Cu copper ion is shown as orange sphere. Selected residues are labeled. Molecular graphics was created using PyMOL (Schrödinger, LLC).

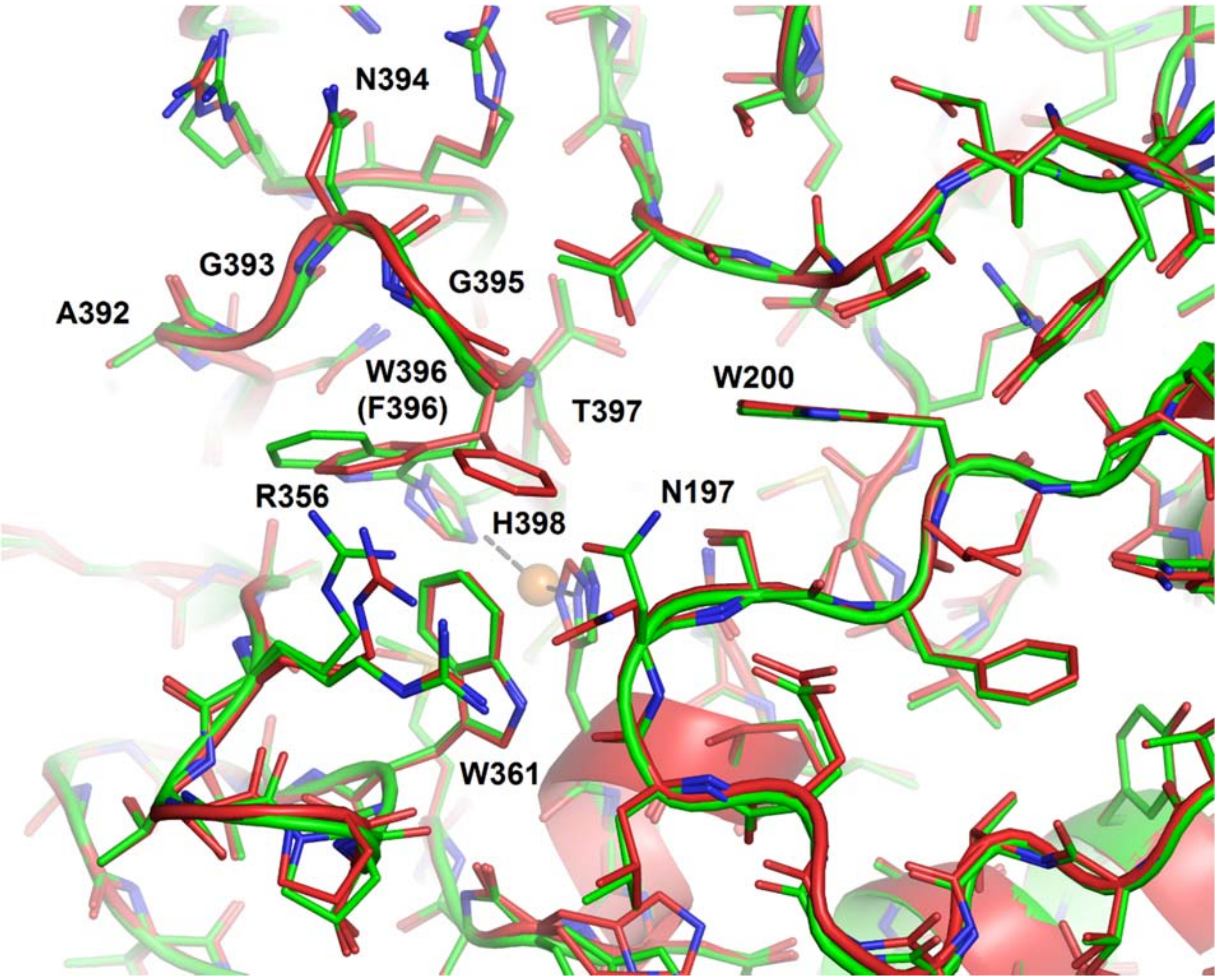

Figure S7. Superposition of the structures *Mv*BOxWT and *Mv*BOxW396F. The mutation of Trp396 to phenylalanine did not affect the structure of the active site. The structure 6I3J of *Mv*BOxWT has carbon colored green, the structure 6I3L of *Mv*BOxW396F red. The T1Cu copper ion is shown as orange sphere. Selected residues are labeled. Notice the disorder of the Phe396 side chain. Molecular graphics was created using PyMOL (Schrödinger, LLC).

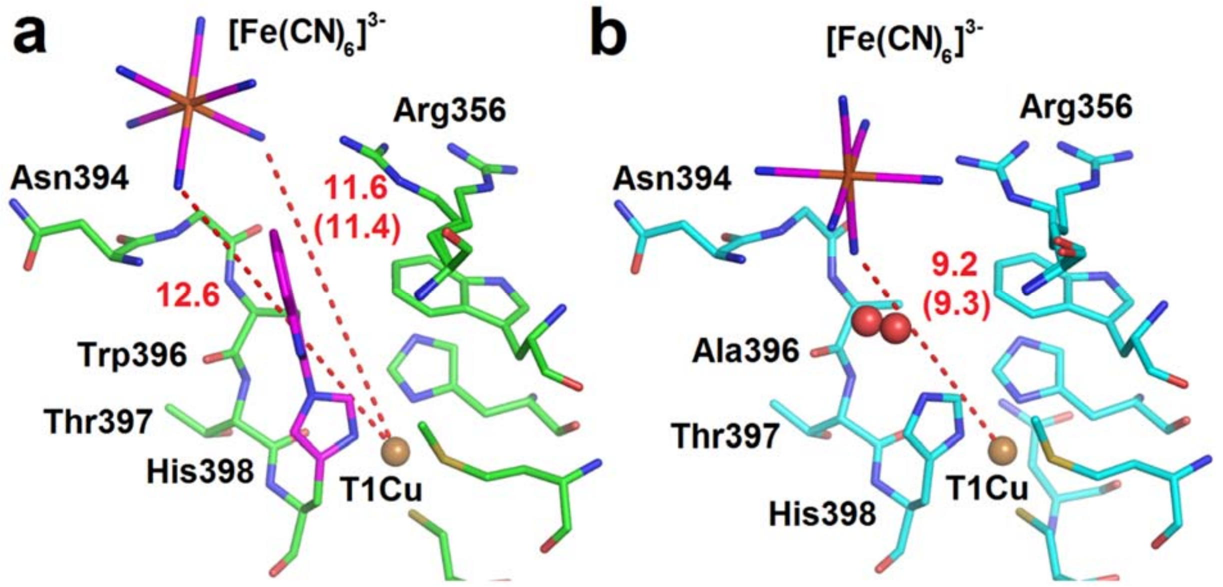

Figure S8. Distance between the bound ferricyanide ion and the T1 copper ion in *Mv*BOxWT and the W396A mutant. (**a**) The structure of WT:FECN (PDB ID 6I3J, carbon green) (**b**) The structure of W396A:FECN (PDB ID 6I3K, carbon light blue). Distances are given in Ångströms. If values differ in chain A and B, they are given in parentheses for chain B of the corresponding structure. Selected residues are labeled. T1Cu is shown as orange sphere. The Trp–His adduct has carbon in magenta, water oxygen is represented by red spheres. Molecular graphics were created using PyMOL (Schrödinger, LLC).

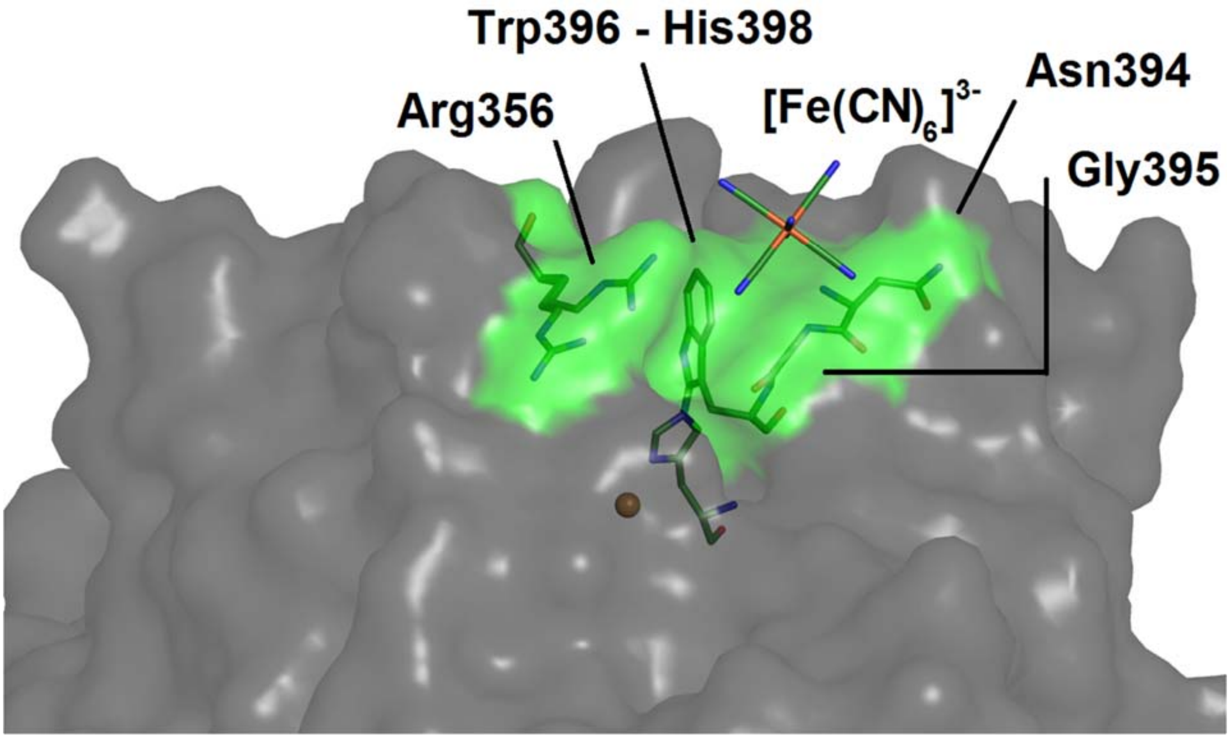

Figure S9. *Mv*BOx surface belonging to oxidation site 1 (OS1). The molecular surface near OS1 is colored grey and the surface of OS1 together with the site-forming residues are colored green. Ferricyanide ion and residues forming OS1 are marked. T1Cu is shown as orange sphere. Molecular graphics was created using PyMOL (Schrödinger, LLC) and the WT:FECN structure (PDB ID 6I3J).

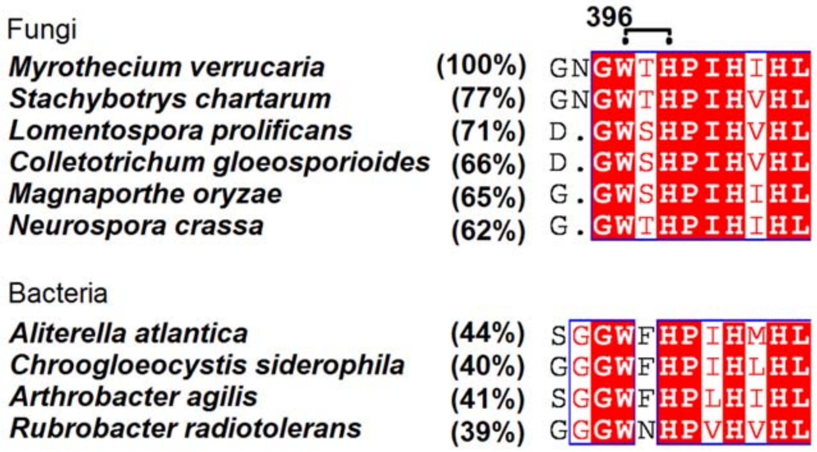

Figure S10. Conservation of residues Trp396 and His398 in homologs of *Mv*BOx from fungi and bacteria. Sequences were identified using NCBI BLAST (3). Only sequences of BOx homologues from a few selected fungi (the first block) and bacteria (the second block) are shown as examples. Sequence identity to *Mv*BOx is shown in parentheses. Graphics was created using ESPript 3 (4).

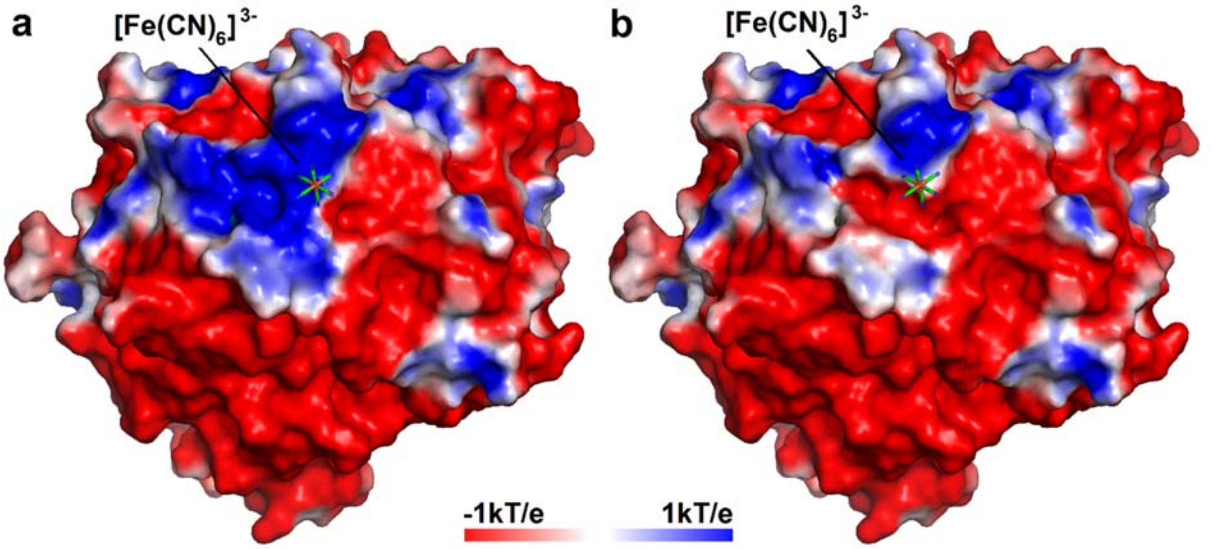

Figure S11. Electrostatic potential distribution of *Mv*BOx represented on solvent accessible surface at pH 7 with ferricyanide ion binding in the oxidation site 1. (**a**) Potential calculated for *Mv*BOxWT using the structure WT:FECN (6I3J). Ferricyanide bound in the active site of *Mv*BOxWT is labeled and shown as sticks with carbon colored green, nitrogen blue, and iron orange. (**b**) Model of *Mv*BOxW396D. The structural model was prepared from the structure of the wild type (6I3J) by replacing Trp396 by Asp and placing it in the conformation with the fewest steric clashes. Ferricyanide is shown in the same position as in the case of WT:FECN (6I3J) to mark the position of the oxidation site 1. The protonation state at pH 7 was assigned by PropKa (5). The parameter file was prepared using PDB2PQR and the AMBER force field (6) and modified to include copper ions. The electrostatic potential distribution was calculated using the linearized Poisson–Boltzmann equation implemented in APBS (7). Molecular graphics was created using PyMOL (Schrödinger, LLC).

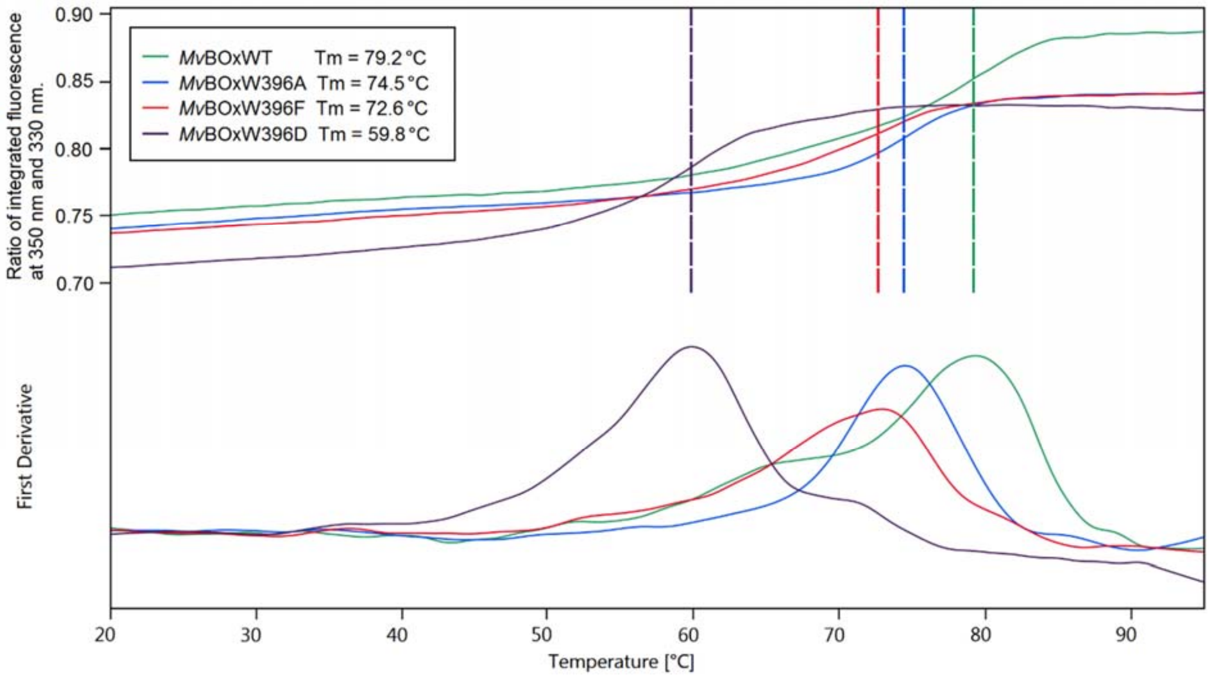

Figure S12. Thermal stability of *Mv*BOxWT and of mutants measured by differential scanning fluorimetry. Measurements were performed using a Prometheus NT.48 (Nanotemper) with protein at concentration 1.5 mg ml^{-1} in 50 mM Tris pH 7.5 and 50 mM NaCl. Measurements were done in temperature range 20-95 °C with rate of temperature increase 2 °C per min.

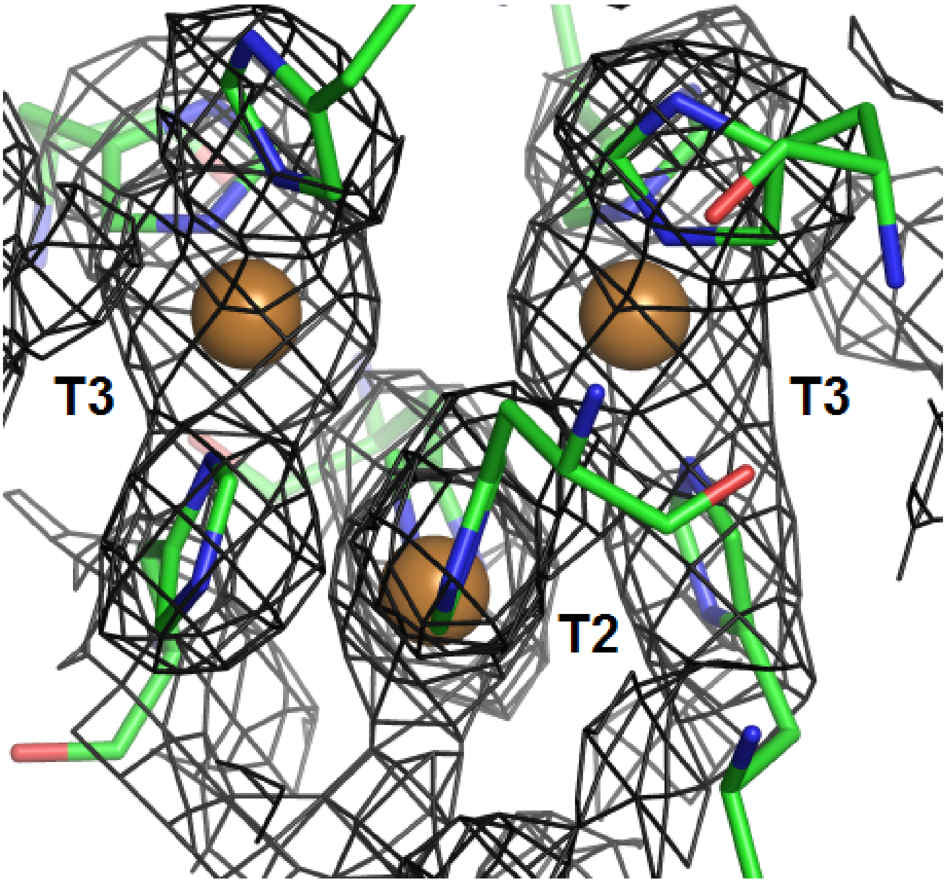

Figure S13. Fully reduced trinuclear cluster in the structure WT:FECN (PDB ID 6I3J). The lack of ligand between T3Cu atoms is in agreement with the reduction of the trinuclear copper cluster in multicopper oxidases. Copper atoms are shown as orange spheres and labeled according to their type. The composite omit electron density map (2m*F*o-D*F*c) is shown as grey mesh and contoured at 1.0 σ. The map was calculated using *Phenix* (8). Molecular graphics was created using PyMOL (Schrödinger, LLC).

REFERENCES

1 Cracknell, J.A., McNamara, T.P., Lowe, E.D. & Blanford, C.F. Bilirubin oxidase from *Myrothecium verrucaria*: X-ray determination of the complete crystal structure and a rational surface modification for enhanced electrocatalytic O_2 reduction. *Dalton Trans*, **40**, 6668–6675 (2011).

2 Akter, M., Tokiwa, T., Shoji, M., Nishikawa, K., Shigeta, Y., Sakurai, T., Higuchi, Y., Kataoka, K. & Shibata, N. Redox potential-dependent formation of an unusual His-Trp bond in bilirubin oxidase. *Chemistry* **24**, 18052–18058 (2018).

3 Boratyn, G.M., Schäffer, A.A., Agarwala, R., Altschul, S.F., Lipman, D.J. & Madden, T.L. Domain enhanced lookup time accelerated BLAST. *Biol Direct*. **7**, 12 (2012).

4 Robert, X. & Gouet, P. Deciphering key features in protein structures with the new ENDscript server. *Nucl. Acids Res*. **42**, 320–324 (2014).

5 Søndergaard, C.R., Olsson, M.H.M., Rostkowski, M. & Jensen, J.H. Improved treatment of ligands and coupling effects in empirical calculation and rationalization of pKa values. *Chem Theor Comput*. **7**, 2289–2295 (2001)

6 Dolinsky, T.J., Nielsen, J.E., McCammon, J.A. & Baker, N.A. PDB2PQR: an automated pipeline for the setup, execution, and analysis of Poisson-Boltzmann electrostatics calculations. *Nucl. Acids Res.* **32**, 665–667 (2004).

7 Baker, N.A., Sept, D., Joseph, S., Holst, M.J. & McCammon, J.A. Electrostatics of nanosystems: application to microtubules and the ribosome. *Proc. Natl. Acad. Sci. USA* **98**, 10037–10041 (2001).

8 Afonine, P.V. *et al.* PHENIX: a comprehensive Python-based system for macromolecular structure solution. *Acta Crystallogr. D Biol. Crystallogr.* **66**, 213–221 (2010).
